# Miscellaneous

**Published:** 1892-01

**Authors:** 


### MISCELLANEOUS.

### BUFFALO LITHIA WATER.

Within the past few years a number of natural springs, strongly impregnated with medicinal properties of great value as remedial agents in various forms of diseases, have been discovered and utilized for the benefit of the afflicted. It would seem as if our good mother NATURE had considerately provided a remedy for every ill "That flesh is heir to," but we are not so instinctively wise as the lower animals in making the discovery. BUFFALO LITHIA WATER, so called from its spring in the "Buffalo Hills" of Virginia, is among the most efficacious in restoring health to the sick and the weary. As a table water it is unsurpassed. Try it.

### CHEMISTRY IN ANCIENT EGYPT.

The priests of Thebes and Memphis made great advances in the knowledge of the art of extracting metals, of forming alloys, and of making vessels and tools out of them. They distinguished crude gold from refined gold, and could work that metal up into a variety of articles. They fed the hope that they might be able to obtain it by coloring asemon or silver yellow. Of the latter metal they made money, the value of which was guaranteed by an impressed image. They extracted gold and silver from electrum, a mineral containing both substances, but which presented to their eyes the appearance of a metal like them. This was what led them to the notion of transmutation.

The Egyptians designated as "chesbet several kinds of blue or green sapphires colored with cobalt or copper. They made incrustations, amulets, necklaces and various ornaments of them. They succeeded in compounding an artificial chesbet resembling the natural stone. A fact worthy of remark in the matter is that this was done by "the assimilation of a colored substance, a precious stone, an enamel, a vitrified color with metals."

This assimilation suggested the new idea of dyeing, for the imitation of the sapphire rests on the coloring of a large mass, colorless by itself, but constituting the vitrifiable basis, which we dye by the aid of a small quantity of coloring matter. With enamels and colored glasses thus prepared the natural precious stones were reproduced. They were covered with figures, with objects of earth or stone, and were incrusted with metallic objects.

Cooked Apples.—Somebody is always telling people how to improve apples for baking by boring out the heart where the rich and fine aroma dwells, and putting in its place some tiresome spice or other. If sound cores, free from worms, can be found, save them for baking. Stewed apples unavoidably lose most of the matchless flavor that is concentrated in the seed core and close under the skin ; for even if the core and skin had not to be removed, the delicate essence they enclose must be mostly driven off by stewing in pieces. Some find a lemon flavoring agreeable in apple sauce ; others do not. The latter, perhaps, will like the result if a little lemon peel and a very small proportion of prunes be added—the lemon, of course, after cooking ; it seems in taste like a reversion of the combined fruits to the pure perfume of their Queen Mother, the Rose. Various other combination tinctures may be formed by experiment. The golden rule of nature is fruits to fruits; but if any one wants nutmeg, etc., let him have it.

An Aluminium Steamboat is now running on the Lake of Zürich, Switzerland. The boat weighs only about half a ton, or about half the weight of an ordinary boat ofthe same size. It was built at Zürich, the metal having been furnished by the aluminium works of Schaffhausen. The boat carries eight persons, and, with a two horse-power petroleum engine, easily makes six miles an hour

### NOSE BLEEDING.

A mild attack of nose bleeding is beneficial, and clears the system as would an attack of illness. In the old or full blooded such an occurrence may stave off an attack of apoplexy. If much blood is lost, or if the attacks are frequent, great prostration will follow, and in some few cases even death has resulted. Slight attacks soon stop of themselves, the escaping blood forming a clot over the bleeding part. It is always wise not to blow the nose or pick it for a day or two after an attack, or another one may come on. A person suffering from nose bleeding should rest and lean forward, so that the blood which escapes may run out of the nostril in front, for if the blood is swallowed it may cause vomiting or sickness. Simple attacks are quickly cured by applying something cold to the spine, as a cold door key or cold stone, as boys stop it. More obstinate cases may have the whole of the spine sponged with cold water, and even the chest and neck as well, the revulsive action of the cold water often stopping it. To insert the fingers up the nostrils and press on the bleeding part is sometimes successful, so also is snuffing cold water up the nostrils ; while some recommend raising the hands above the head to stop it. If very great loss occurs it will be wise to call in a doctor, who will plug up the nostrils if he thinks it necessary.

### GOOD MANNERS AT HOME.

The presence of good manners is nowhere more needed or more effective than in the household, and perhaps nowhere more rare. Whenever the familiarity exists, there is a tendency to loosen the check upon selfish conduct which the presence of strangers involuntarily produces. Many persons who are kind and courteous in company, are rude and careless with those whom they love best. Emerson says : " Good manners are made up of pretty sacrifices," and certainly nothing can more thoroughly secure the harmony and peace of the family circle than the habit of making small-sacrifices one for another. Children thus learn good manners in the best and most natural way, and habits thus acquired will never leave them. Courtesy and kindness will never lose their power or their charm, while all spurious imitations of them are to be despised.

### A CHEAP BINDING.

A correspondent of the *Scientific American* says : " I have bound twenty volumes in this way : Pack the papers smoothly, hold firmly, and drive a thin chisel through the pile about half an inch from the back. Push a strong tape through and leave about two inches ; put three or four tapes through at even intervals ; cut common thick paper boards large enough to project a little everywhere, except that one edge must come front of the tapes ; draw the tapes tightly and glue down to the boards outside; skive a piece of leather—common sheepskin will answer—wide enough to cover the back and come on the boards an inch or two, and long enough to project a couple of inches at the end ; paste the leather well ; put it on the back; fold the ends in so as to come over the boards on each side; paste any fancy or plain paper over the sides, and, lastly, paste the blank leaf down to the cover side, and you have a presentable book, and very durable. Trimming the edges can be easily done by clamping between boards and cutting the edges with a thin, sharp knife by a straight edge. Of course, this is done before the boards are put on, after the tapes are in. This makes a flat-edge book, but for a thin book answers very well.

### GIRLS' SHOES.

I lay great stress upon the construction of the shoe. It should not be coarse and heavy, as then it will be a constant source of mental irritation to the wearer; for girls, even at this age, are beginning to take pride in a comely foot, and is a pride to be fostered with a kindly care. The foot, with a little forethought, can be properly attired, and at the same time be both healthfully and neatly dressed. I take this opportunity to say a word in condemnation of that modern abomination to womenly grace and comfort, the French heel. These heels displace the supporting base of the body by forcing upward the keystone of the arch on which it rests, thereby weakening the whole super-structure. Their injurious effects are not confined to the feet—their baneful influence pervades the whole economy.

Outdoor Exercise.—Take as much exercise as you can, and be in the open air as much as possible. Outdoor life is the natural condition of mankind and the more one can have of it the better. The practice must not be carried to extremes, however. There are many days when one is much better off in a warm, comfortable, well ventilated house than trying to take out-door exercise in a mid-winter storm, or under a July sun.

